# Gluten-Free Diet Indications, Safety, Quality, Labels, and Challenges

**DOI:** 10.3390/nu9080846

**Published:** 2017-08-08

**Authors:** Kamran Rostami, Justine Bold, Alison Parr, Matt W. Johnson

**Affiliations:** 1 Department of Gastroenterology, Milton Keynes University Hospital, Milton Keynes MK6 5LD, UK; kamran.rostami@nhs.net; 2 Allied Health and Social Sciences, Institute of Health and Society University of Worcester, Worcester WR2 6AJ, UK; 3 Freelance Nutrition Therapist, Manchester M33 5PD, UK; alison_parr@hotmail.com; 4 Department of Gastroenterology, Luton and Dunstable University Hospital, Luton LU4 0DZ, UK; matthew.johnson@ldh.nhs.uk

**Keywords:** gluten-free diet, coeliac disease, non-coeliac gluten sensitivity

## Abstract

A gluten-free diet (GFD) is the safest treatment modality in patient with coeliac disease (CD) and other gluten-related disorders. Contamination and diet compliance are important factors behind persistent symptoms in patients with gluten related-disorders, in particular CD. How much gluten can be tolerated, how safe are the current gluten-free (GF) products, what are the benefits and side effects of GFD? Recent studies published in *Nutrients* on gluten-free products’ quality, availability, safety, as well as challenges related to a GFD are discussed.

## 1. Editorial

**Figure 1:**
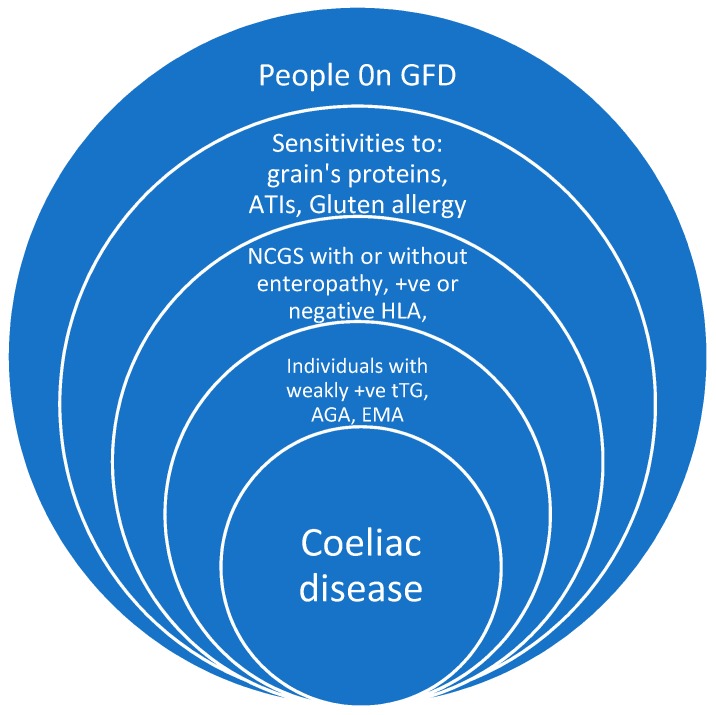
Gluten-related disorder, tTG: Tissue transglutaminase antibodies, AGA: Antigliadin antibodies, EMA: Endomysial antibodies, NCGS: Non-coeliac gluten sensitivity, ATIs: Amylase/trypsin inhibitors.

Gluten-free diets hit centre stage in the early 1990s, and universally changed our food culture. Not only was there interest in coeliac disease (CD) [1], but there was also a resurgence of interest in the other gluten-related disorders [2,3]. Current evidence suggests that gluten and other wheat proteins play an important role in triggering symptoms in some people without CD [4]. There has been a rapid increase in dietary interest to use it as a treatment modality in the management of both irritable bowels syndrome (IBS) and functional bowel disorders. This strategy has evolved as a result of improvements in our understanding of how these grains induced pathogenicity [5,6]. The grains that contain gluten seem to have the potential of antigenicity, relating not only to the gluten itself [7] but also to their other proteins and additives. Junker et al. suggest that α-amylase/trypsin inhibitors (ATIs) in wheat represent strong activators of innate immune responses in monocytes, macrophages, and dendritic cells [8]. Therefore, a large proportion of the world’s population is currently avoiding gluten-containing grains for a variety of different reasons; including sensitivities, intolerances, and allergic reactions (Figure 1).

Gluten intake, in particular prolamin, is a well-known triggering antigen that initiates adaptive T cells (Th1-mediated) immune response in individuals carrying HLA-DQ2 or HLA-DQ8 against small bowel cells. This in turn leads to an architectural distortion in CD. Epithelial cell damage is the first event to occur within the small bowel, leading to antigen increased intestinal permeability and malabsorption (even in the absence of severe inflammation). Some studies suggest gluten may affect diabetes development by influencing proportional changes in immune cell populations or by modifying the cytokine/chemokine pattern towards an inflammatory profile. Gluten-induced intestinal inflammation might in fact play a primary role in the pathogenesis of type 1 diabetes, by islet-infiltrating T cells expressing gut-associated homing receptors [9]. This is why untreated CD increases the risk for other autoimmune disorders and long-term complications.

Gluten cannot be hidden in foods nowadays, as allergen labelling was introduced in the European Union (EU) in 2005. Now all wheat, rye, barley, and oat ingredients must be listed in the ingredients list. The amount of gluten capable of initiating an antigenic reaction has been estimated to be >20 mg/kg (or parts per million = ppm) of gluten, and contamination below 20 ppm is considered safe over a wide range of foods in daily consumption.

The EU gluten-free legislation published in 2009 and regulated in 2012 specifies two levels—gluten-free (≤20 ppm/mg/kg) and low gluten (21–100 ppm/mg/kg), but in practice only the gluten-free standard is applied. If products do not have any gluten-containing ingredients, then an associated threshold would not be necessary.

## 2. How Do Gluten-Free Products Compare to a Normal Diet?

Coeliac disease was a difficult diagnosis to live with 20 years ago. This was largely due to the limited range of gluten-free products available to coeliac patients as well as the generally poor quality of these products.

Over recent years, the gluten-free food business has become a major industry and has gained enormous popularity with both coeliac and non-coeliac individuals, as there have been improvements in both the range of foods available and their overall palatability. Despite some concerns about potential side effects of GFD, the current evidence suggests that there is no need to be concerned as long as it proves beneficial in controlling symptoms and improving the quality of life. Some individuals consider GFD to be a very balanced and healthy diet, especially if gluten-free wholegrains are consumed, whilst others find it useful for weight control due to its restrictive nature.

GFD is not recommended for the general population, and there is no evidence that it is beneficial in non-symptomatic non-coeliac individuals. Instead, there are some concerns raised relating to its nutritional value. Recent studies suggest that GFDs might be a risk factor for metabolic syndrome [10]. They state that the nutritional composition of processed gluten–free food items may include high levels of lipids, sugars, and salt [11]. Saturni et al. report that a gluten-free diet may not guarantee adequate nutritional intake and that 20–38% of coeliac patients experience nutritional deficiencies that include proteins, dietary fibres, minerals, and vitamins [12]. It is unclear if persistent malabsorption syndrome accounts for some part of these deficiencies. For instance, secondary lactose intolerance might be one of the factors contributing toward vitamin D deficiency in CD [13].

Contrary to this, gluten-free substitute foods are not necessarily higher in sugar or lower in fibre according to Coeliac UK. There have been improvements in the quality of gluten-free products, such as the development of a broader range of fresh gluten-free items as well as products higher in fat, increasing palatability. There is, however, still room for improving the nutritional value and component qualities of gluten-free products.

It should be remembered that any treatment modality, including diet, might potentially have undesirable effects. Furthermore, this information, when available, should be highlighted to potential candidates [14]. If we compare the side effects of GF products with the drugs licenced to treat irritable bowel syndrome (IBS) and their enormous cost, a treatment with GFD is much less toxic and patients are able to tailor their personal dietary preferences with a little guidance [15]. Education is a key factor in achieving a much healthier dietary balance. A dietetic consultation or any consultation where food and diet are discussed should not entirely focus on the elimination of gluten, but also provide guidance for healthy choices based on the individual’s needs.

Health professionals should also be mindful that a high sugar and fat content are not only found in some GF products, but are common in many other foods as well, indicating that the general population not on GFD is also exposed to a wide range of high calorie products and risks for metabolic syndrome. Ultimately, genetics and lifestyle factors, regardless of gluten intake, have the most significant influence in preventing or acquiring morbid obesity. Health professionals are most likely aware that the prevalence of obesity is unfortunately increasing in both coeliac and non-coeliac populations [16,17]. Once again, this highlights how education and the promotion of a healthier lifestyle is a public health priority for both the general population and gluten-sensitive individuals.

## 3. Cost and Availability

Gluten-free now constitutes a major food industry due to its popularity among not only CD patients but also individuals with other gluten-related conditions (Figure 1). The cost of living for patients following a GFD is much higher and it is quite challenging for people with a lower income to purchase the products without government support.

The products available to some in the UK on prescription may also not be easily available for purchase off-the-shelf, and it should be noted there is now a trend for GF prescription to be withdrawn, indicating that some patients are affected due to the lack of affordability. From a patient’s perspective, the availability of GF products does not always square with accessibility, i.e., being at the shop shelf at the point of need. The government’s support with prescription products has been a significant encouragement and support for patients, and withdrawing the prescription for the limited amount of permitted, prescribed food may result in reduced diet compliance, leading to increased complications and higher expenses for the healthcare system in the long term.

## 4. Safety and Contamination

Despite the availability of numerous GF products in the current market, maintaining a GFD is still challenging for many patients. Therefore, dietary transgressions of GFD is a major factor for refractory symptoms and persistently abnormal histology. Still, it should be noted that recently there have been substantial improvements in the commercial availability of a variety of GF products that offer a wide range of choices to gluten-sensitive people. Reassuringly, a recent study demonstrates that the safety profile of these products is improving thanks to quality assurance legislations. This recent study published in *Nutrients* analyses the contamination risk and changes in the gluten content of an impressive number of GF products (3141) from 1998 to 2016 [18,19]. The former is one of the largest published studies presenting data from food samples collected over an 18-year period.

The time period covers a lot of change in terms of the standard for gluten-free products in addition to the introduction of different methodologies for the analysis of gluten-free products, which may help to explain some of the idiosyncrasies in the data analysis and reporting over the period. The Codex standard for gluten-free changed in 2008, and was prefaced by agreement on the R5 Mendez method for the analysis of gluten in 2005–2016, endorsed by the Codex Committee on Methodology and Analysis. Eight useful food categories are identified, and the authors demonstrate that cereal-based foods for people with CD are becoming safer.

It is, however, concerning that in the period of 2013–2016 there were increases in the number of white flour samples with gluten contamination at 100 mg/kg—as this is such a staple food ingredient in gluten-free baking, contamination at this level can be problematic. Two different ELISA analyses were used in the study to determine the level of gluten contamination across the period of 1998–2016—one method was used from 1998–2001 and a different method was used from 2001–2016. The R5 ELISA Mendez method is mentioned as the type one methodology for the analysis of gluten in foodstuff in the Revised Codex Alimentarius standard (2008), but not in the EU legislation. Whilst both methods are recommended by the Codex Alimentarius and AOAC international, the reporting periods in the study do not mirror these time periods. As three periods have been used: 1998–2002, 2003–2008, and 2009–2016, perhaps it might have been beneficial to report particularly on the 1998–2001 period itself given that this period used a different ELISA technique.

The Italian study [19] includes 200 certified GF foods and many foods that are naturally gluten-free—such as buckwheat, quinoa, etc. These are rarely included in other studies, and there is a paucity of data on these products in the current literature. Therefore, assessing the safety of these products is another valuable part of this review that may improve the nutritional quality and the experience of a GF diet for gluten-sensitive individuals. The benefits of these products, as highlighted by the authors, is very informative for any professional giving dietary advice. particularly as they are wholegrain cereals, such food products are welcome additions to a GFD given the concerns about the high sugar content of some GF foods. The study provides statistical analysis, consideration of factors such as cost, and a categorisation of foods into types (and meal types). The Italian study reports some important findings, namely that four out of five oat samples tested were contaminated with gluten, as were several sample of buckwheat and lentils (the latter was unexpected and the authors state that the origin of the contamination is unknown). Sadly, the authors do not specify the individual brands, or whether the samples were certified as GF. Lunch and dinner foods were more contaminated compared to snack products. Hence, professionals working with coeliac patients should consider highlighting the importance of buying certified gluten-free oats and oats-based products.

Studies such as those mentioned above show the importance of ongoing regulation and control of certified GF foods as well as the importance of on-going policing of those foods. Interestingly, they show that cheaper foods have higher contaminations, suggesting that better control costs more. this indicates that patients with lower incomes might be exposed to a higher risk for contamination, particularly as many gluten-free prescriptions in the UK are now under threat.

## 5. Conclusions

Gluten, additives, and a range of other grain proteins have all been associated with a range of gastrointestinal and autoimmune disorders, particularly CD. Despite the concerns related to the content of some GF products, this modality of treatment is still the safest strategy available to coeliac patients and other gluten related disorders (GRD) patients. The concerns related to metabolic syndrome should not be limited to GFD and should include the modern lifestyle. Transgression related to high contents of gluten in GF products has been a culprit for refractory symptoms in CD patients. Reassuringly, a recent study has suggested that gluten contamination is uncommon or mild; however, it is occurring and needs both on-going and tighter regulation in order to protect those people who are sensitive to gluten.
